# Development and validation of the osteoporosis scale among the system of quality of life instruments for chronic diseases QLICD-OS (V2.0)

**DOI:** 10.1186/s12877-024-05019-9

**Published:** 2024-05-07

**Authors:** Qiongling Liu, Lie’e Li, Wanrui Ma, Zheng Yang, Rui Zhao, Caixia Liu, Chonghua Wan

**Affiliations:** 1 The First Dongguan Affiliated Hospital of Guangdong Medical University, Dongguan, China; 2 School of Nursing, Key Laboratory of Lifecycle Care for Chronic Diseases, The Fundamental Nursing Research Institute, Guangdong Medical University, Dongguan, 523808 China; 3 Teaching Management Office of The First Affiliated Hospital of Shenzhen University, Shenzhen, 518000 China; 4 Research Center for Quality of Life and Applied Psychology, Key Laboratory for Quality of Life and Psychological Assessment and Intervention, Guangdong Medical University, Dongguan, 523808 China; 5 Department of Nursing, Henan Cancer Hospital, Zhengzhou, 450008 China; 6 Department of Rehabilitation, Guangzhou Hospital of Integrated Traditional Chinese and Western Medicine, Guangzhou, 510800 China

**Keywords:** Osteoporosis, Quality of life, The disease-specific module, Psychometric properties

## Abstract

**Background:**

Quality of life of osteoporosis patients had caused widespread concern, due to high incidence and difficulty to cure. Scale specifics for osteoporosis and suitable for Chinese cultural background lacked. This study aimed to develop an osteoporosis scale in Quality of Life Instruments for Chronic Diseases system, namely QLICD-OS (V2.0).

**Methods:**

Procedural decision-making approach of nominal group, focus group and modular approach were adopted. Our scale was developed based on experience of establishing scales at home and abroad. In this study, Quality of life measurements were performed on 127 osteoporosis patients before and after treatment to evaluate the psychometric properties. Validity was evaluated by qualitative analysis, item-domain correlation analysis, multi-scaling analysis and factor analysis; the SF-36 scale was used as criterion to carry out correlation analysis for criterion-related validity. The reliability was evaluated by the internal consistency coefficients Cronbach’s α, test-retest reliability Pearson correlation r. Paired t-tests were performed on data of ​​the scale before and after treatment, with Standardized Response Mean (SRM) being calculated to evaluate the responsiveness.

**Results:**

The QLICD-OS, composed of a general module (28 items) and an osteoporosis-specific module (14 items), had good content validity. Correlation analysis and factor analysis confirmed the construct, with the item having a strong correlation (most > 0.40) with its own domains/principle components, and a weak correlation (< 0.40) with other domains/principle components. Correlation coefficient between the similar domains of QLICD-OS and SF-36 showed reasonable criterion-related validity, with all coefficients r being greater than 0.40 exception of physical function of SF-36 and physical domain of QLICD-OS (0.24). Internal consistency reliability of QLICD-OS in all domains was greater than 0.7 except the specific module. The test–retest reliability coefficients (Pearson r) in all domains and overall score are higher than 0.80. Score changes after treatment were statistically significant, with SRM ranging from 0.35 to 0.79, indicating that QLICD-OS could be rated as medium responsiveness.

**Conclusion:**

As the first osteoporosis-specific quality of life scale developed by the modular approach in China, the QLICD-OS showed good reliability, validity and medium responsiveness, and could be used to measure quality of life in osteoporosis patients.

## Introduction

Osteoporosis is a chronic metabolic bone disease [1]. At present, about 200 million people worldwide suffer from osteoporosis. Its incidence has jumped to the 7th place among common and frequently-occurring diseases [2]. China has the largest elderly population in the world. It is estimated that by 2050 the number of osteoporosis patients in China would reach 212 million [3]. A new study conducted by the Osteoporosis Foundation shows that the total prevalence of osteoporosis in China is 6.6-19.3%, with an average of 13% [4]. One-third of osteoporosis patients are disabled with 19% of them requiring long-term care. Compared with the general population, patients with osteoporosis had more challenges in physical and mental health. While suffering from the disease, patients with osteoporosis also had to bear financial pressure, adverse drug reactions brought about by anti-osteoporosis drug treatment, psychological burden caused by family neglect and decline in social function. Therefore, the loss of labor function, disability, mental pain and the corresponding psychological burden caused by osteoporosis to patients had severely affected their quality of life (QOL) [5].

The premise and key of Quality of Life research was the appropriate measurement scale, which mainly included the generic scale and the specific scale. The generic scale could be used for the general population and multiple disease groups to assess general health status. Although the prevalence of different diseases could be directly compared with this type of scale [6, 7], it ignored the main functions affected by the disease and led to the loss of clinically important influencing factors. Thus, the responsiveness was poor when used for specific diseases. Disease-specific scales had the advantage of assessing domains related to specific diseases and capturing the sensitivity of small changes [6, 7]. As far as we knew, some major foreign specific scales currently include Osteoporosis Quality of Life Questionnaire (OQLQ) [8], Japanese Osteoporosis Quality of Life Questionnaire (JOQLQ) [9, 10], Osteoporosis Assessment Questionnaire(OPAQ) [11], Osteoporosis Functional Disability Questionnaire (OFDQ) [12], Quality of Life Questionnaire of the European Foundation for Osteoporosis (QUALEFFO) [13] and Assessment of health related quality of life in osteoporosis (ECOS-16) [14, 15]. OPAQ was the first special scale for osteoporosis compiled in 1993. It contained 79 items in four aspects, i.e. symptom, physical, psychological, and social conditions. It was mainly used in patients with non-vertebral fractures. QUALEFFO was developed by the European Foundation for Osteoporosis and included 48 items in five aspects, covering pain, physical function, social function, general health concepts, and psychological factors. It was mainly used to evaluate vertebral fracture patients with severe osteoporosis. JOQLQ was developed in Japan and contained 38 items in six aspects, covering pain, activities of daily living, entertainment and social activities, general health, posture and body shape, falls and psychological factors. It was used to assess the quality of life of Japanese osteoporosis patients. ECOS-16 contained 16 items in four aspects, and was mainly used to evaluate postmenopausal women with osteoporotic vertebral fractures. There was a special scale for the quality of life of primary osteoporosis compiled by Jian Liu in China [16, 17]. According to Liu, OQOLS was mainly used to assess patients with primary osteoporosis, including 75 items in five aspects, i.e. symptoms, physiology, psychology, society, and satisfaction. This scale did not involve the evaluation of adverse drug reactions and special psychological problems of the disease. The scales mentioned above were developed independently and lacked systematic coherence. In addition, they may not reflect Chinese culture well. Therefore, it was necessary to develop a scientific, reasonable, reliable and suitable quality of life measurement scale for Chinese osteoporosis patients.

To this end, our QOL team developed a system entitled Quality of Life Instruments for Chronic Diseases (QLICD), which included a general module (QLICD-GM), and some specific modules for different diseases [18, 19]. The latest version of the system QLICD (V2.0) contained 34 chronic disease-specific scales [19], including QLICD-CG for Chronic Gastritis [20], QLICD-PT for Pulmonary Tuberculosis [21], QLICD-RA for Rheumatoid Arthritis [22] and QLICD-SLE for Systemic Lupus Erythematosus [23] etc. Among them, QLICD-OS (Quality of Life Instruments for Chronic Diseases-Osteoporosis) was developed by combining the general module of chronic diseases and the specific module of Osteoporosis, with the purpose to suit for osteoporosis patients under Chinese cultural background. It was both specific and comparable (comparing common parts of various diseases).

This article aims to report the development and validation process and results of QLICD-OS (V2.0).

## Methods

### Development of QLICD-OS

QLICD-OS was compiled by combining the general module of chronic diseases QLICD-GM [18, 19], and the newly developed osteoporosis disease-specific module.

### Development of QLICD-GM

The development of the QLICD-GM (V2.0) strictly followed the internationally recognized method of programmatic decision-making, including the following steps: (1) Established a scale research team; (2) Defined and decomposed the concept of quality of life measurement to form a theoretical framework; (3) Proposed a pool of alternative items; (4) Screened items to form a preliminary scale; (5) Conducted pre-survey item screening to form a test scale; (6) Test survey and item re-screening; (7) Scale evaluation; (8) Formed a formal scale.

**Fig. 1:**
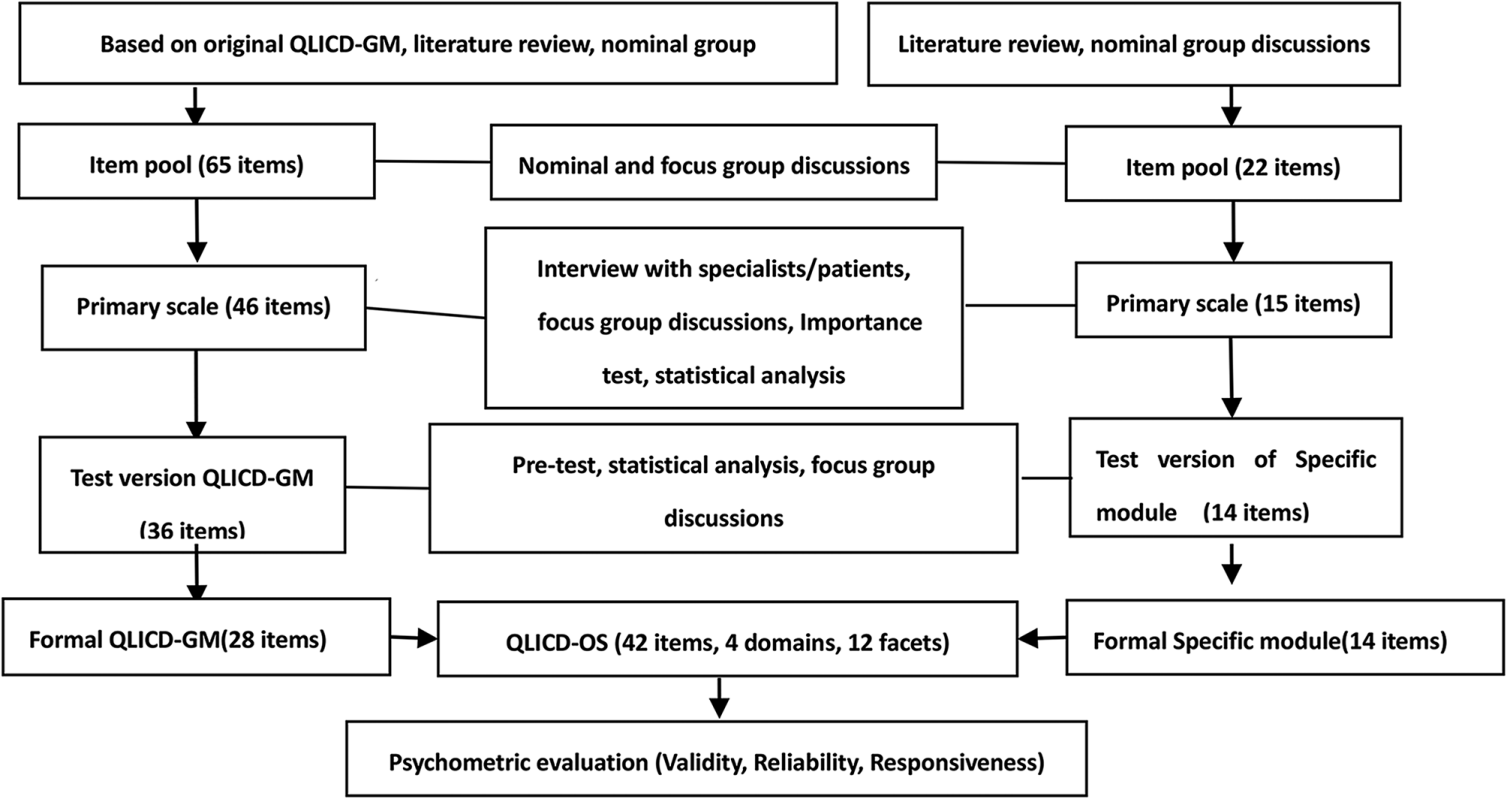
Steps towards development and validation procedure of QLICD-OS

In the end, QLICD-GM (V2.0) was developed, including 3 domains which were physical function (9 items), mental function (11 items), and social function (8 items) and 9 facets, a total of 28 items (See Fig. 1 in detail).

### Development of osteoporosis specific module

Similar to QLICD-GM [18, 19] and other specific modules for hypertension, coronary heart disease and peptic ulcers [24–26], the osteoporosis disease-specific module was completed through the efforts of two independent groups. The nominal group consisted of 14 people, including 5 doctors, 2 nurses, 2 medical educators, and 5 teachers/researchers (1 quality of life researcher, 1 statistician, 1 sociologist, and 2 psychologists), which proposed the item pool using programmatic decision-making method. The focus group was composed of 10 experts, including 4 doctors, 1 medical educator, and 5 teachers/researchers (2 quality of life research scholars, 1 statistician, 1 sociologist, and 1 psychologist), which proposed the conceptual framework using programmatic decision-making method and selected items proposed by the nominal group. In general, the nominal group was responsible for item presentation, while the focus group was responsible for item selection and organization. In the item selection process, both qualitative analysis methods such as group discussions, in-depth interviews as well as quantitative statistical methods for pre-tests data such as variation analysis, correlation analysis, and factor analysis were used.

The scale was developed based on the literature review, nominal group/focus group discussion, and the experience of setting up the scale at home and abroad. The 22-item pool of the osteoporosis disease-specific module was initially screened, evaluated and modified through a combination of qualitative interviews and quantitative investigation and analysis to form a preliminary scale. Questionnaire surveys and interviews were conducted on osteoporosis patients and medical experts, including 25 patients and 25 doctors/ nurses. The data were analyzed using variability method, correlation coefficient method, factor analysis, patient importance scoring and doctor importance scoring.

In the end, the final specific module was formed including 3 facets of clinical symptoms (CLS), drug side effects (DSE), and special Effects on Mentality and Life (EML) of osteoporosis, and a total of 14 items (coded as OP1-OP14) [27], (See Fig. 1 in detail) .

The entire development and evaluation process was summarized in Fig. 1.

### Validation of QLICD-OS

Based on the measured data scores, the measurement characteristics of QLICD-OS were evaluated from the perspectives of validity (construct validity and content validity), reliability (internal consistent reliability and test-retest reliability), and responsiveness [28].

### Data collection

Similar to other instruments under the system of QLICD [18–26], the QLICD-OS scale was designed particularly suitable for the Chinese population and was used for on-site investigation and evaluation of patients with osteoporosis. The survey was conducted at Pingle Orthopedics Hospital in Shenzhen, Guangdong Province, China. The research objects were osteoporosis patients with certain reading comprehension ability and ability to fill out the questionnaire independently. The investigators in the research include doctors/nurses and medical graduate students. The investigators explained the purpose and significance of the study to the patients, and obtained the informed consent of the patients who agreed to participate in the study. The research protocol and informed consent form were approved by the Ethics Committee of the survey institution.

In the first round of assessment process, each subject (*n* = 127) completed a questionnaire when he or she was admitted to the hospital for treatment. On the 2nd day, some respondents (*n* = 117) were selected to participate in the second round of assessment for test-retest reliability. After one week of treatment, a total of 127 subjects participated in the third round of assessment for responsiveness assessment.

Due to the lack of a recognized gold standard for assessing the quality of life of patients with osteoporosis, we used the Chinese version of the 36-item Health Measurement Scale (SF-36) [29] for evaluation of the criterion-related validity as well as convergent and discrimination validity of QLICD-OS at first round. SF-36 was considered one of the commonly used universal QOL scale, including 8 dimensions: Physical function (PF), role physical (RP), body pain (BP), general health (GH), vitality (VT), social function (SF), role of emotion (RE), and mental health (MH).

### Scale scoring method

Similar to other instruments under the system of QLICD [18–26], each item of QLICD-OS was scored based on the five-level Likert scale (namely, not at all, a little bit, somewhat, quite a bit, and very much). The positively stated items directly scored from 1 to 5, while reverse entries were scored from 5 to 1. The higher the score of the positive item, the higher the quality of life, and the opposite is true for the reverse item. Specifically, GPH1, GPH2, GPH4, GPH6, GPH7, GPH8;GPS1, GPS3, GPS10; GSO1, GSO2, GSO3, GSO4, GSO5, GSO8 are positively stated items, and the others are negatively stated items. The content of items can be found in item brief description in relevant table.

By adding up the domain/facet item scores, we obtained the raw scores of facets and domains. The total score of the scale was the sum of the scores in all domains. For comparison, the following equation was used to linearly convert all domain scores into standardized scores (SS) between 0 and 100: SS=(RS-Min)×100/R, where RS, Min, and R represented the original score, the lowest score, and score range.

### Validity evaluation

There are several types of validity that can be distinguished. The content validity adopted a qualitative evaluation method. Due to the lack of gold standard, SF-36 scale was used as the criterion and Pearson correlation coefficient between similar domains of QLICD-OS and SF-36 was calculated to evaluate the criterion-related validity. Gerry believed that the ideal correlation coefficient was between 0.4 and 0.8 [30]. Multi-trait scaling analysis [31] was applied to test the convergent and discrimination validity of QLICD-OS, which was an aspect of the construct validity. It has the following two standards: (1) item-domain correlation which was 0.40 or higher supported the convergent validity; (2) item-domain correlation which was higher than other domains supported the discrimination validity.

Also the factor analysis with Varimax rotation was adopted to perform to test the consistency between the components extracted from the data and the theoretical structure of the scale, confirming the construct validity.

### Reliability evaluation

Reliability refers to the degree to which the instrument is not affected by random errors and is evaluated by internal consistency and repeatability. Cronbach’s α was a common method to assess the internal consistent reliability in the scale development. Coefficient between 0.6 and 0.7 was the minimum acceptable value, coefficient between 0.7 and 0.8 was quite good, and coefficient value between 0.8 and 0.9 was very good [32]. In order to evaluate internal consistency, Cronbach’s α for each domain was calculated separately.

Test-retest reliability for the QLICD-OS was assessed using correlation *r* with the threshold being recognized as 0.80.

### Responsiveness evaluation

Responsiveness referred to the ability of the scale to detect small clinically important changes over time [28, 33, 34]. Responsiveness was measured by comparing the average difference between pre-treatment and post-treatment assessments. Meanwhile, standardized response mean (SRM) was calculated to represent the degree of responsiveness, and 0.20, 0.50 and 0.80 represented small, medium, and large responsiveness respectively [28, 33, 34].

## Results

### Demographic characteristics of the sample

The age of 127 patients with osteoporosis ranged from 33 to 84 years old with an average age of 66.55 years. 103 female participants (81.10%), and 122 participants (96.06%) were Han nationality. Most participants (91 cases, 71.65%) were married and 32 cases (25.20%) were widowed. In terms of educational level, 47 participants (37.01%) graduated from primary school, 32 (25.20%) graduated from secondary school, 22 (17.32%) graduated from high school or technical secondary school, and 26 (20.47%) graduated from college or university. Among them, 38 (29.92%) were farmers, 14 (11.02%) were teachers, and 17 (13.39%) were administrative personnel. Most of the participants (*n* = 80, 62.99%) were under social medical insurance.

### Validity

#### Content validity

Content validity referred to whether the designed item could represent the content or topic to be measured. QLICD-OS was compiled according to a strict procedural method with the items of the scale including all the dimensions required by WHO QOL group. Also QLICD-OS was developed after repeated discussions by the nominal group and the focus group, which included aspects of physical, psychological, social condition and clinical symptoms, drug side effects, and special psychological characteristics of patients with osteoporosis. These aspects fully reflected the connotation of the quality of life of patients.

#### Construct validity

From correlation analysis, it can be seen that there were sufficiently associations between items and their own domains to which they belonged, but weak associations between items across domains (Table 1). For example, most correlation coefficients between items of GPH1-GPH9 with physical function (in bold) are greater than 0.4, and greater than those across domains.

**Table 1:** Correlations between items and domains of QLICD-OS for osteoporosis patients

Item code	Item brief description	Physical function	Mental function	Social function	Specific module
GPH1	Appetite	0.59**	0.45**	0.47**	0.15
GPH2	Sleep	0.45**	0.35**	0.32**	0.16
GPH3	Sexual function	**0.08**	0.13	0.11	0.01
GPH4	Excrement	0.54**	0.31**	0.34**	0.24**
GPH5	Pain	0.34**	0.36**	0.47**	0.22**
GPH6	Daily activities	0.88**	0.45**	0.56**	0.21*
GPH7	Work	0.83**	0.40**	0.46**	0.24**
GPH8	Walk	0.89**	0.51**	0.55**	0.24**
GPH9	Fatigue	0.49**	0.52**	0.38**	0.28**
GPS1	Attention	0.47**	0.46**	0.35**	0.30**
GPS2	Memory deterioration	0.22**	0.42**	0.28**	0.39**
GPS3	Joy of life	0.54**	0.58**	0.55**	0.13
GPS4	Restless	0.44**	0.62**	0.43**	0.19*
GPS5	Family burden	0.46**	0.71**	0.61**	0.16
GPS6	State of health	0.30**	0.70**	0.39**	0.31**
GPS7	Depression	0.40**	0.77**	0.42**	0.19*
GPS8	Disappointment	0.22**	0.71**	0.31**	0.15
GPS9	Fear	0.38**	0.70**	0.51**	0.27**
GPS10	Positive attitude	0.49**	0.68**	0.58**	0.09
GPS11	Termagancy	0.35**	0.65**	0.40**	0.20*
GSO1	Social contact	0.59**	0.54**	0.79**	0.17*
GSO2	Family relationship	0.32**	0.44**	0.66**	0.01
GS03	Friend relationship	0.48**	0.47**	0.80**	0.06
GSO4	Family support	0.42**	0.49**	0.74**	0.09
GSO5	Other people’s care	0.45**	0.42**	0.78**	0.06
GSO6	Economic hardship	0.17*	0.46**	0.36**	0.12
GSO7	Labor status	0.30**	0.42**	0.57**	0.08
GSO8	Family role	0.58**	0.50**	0.69**	0.26**
OS1	Lower back pain	0.35**	0.35**	0.21*	0.45**
OS2	Bone/joint pain	0.10	0.03	0.04	0.40**
OS3	Cramps	0.16	0.07	0.11	0.56**
OS4	Getting shorter	0.40**	0.31**	0.29**	0.58**
OS5	Prone to fractures	0.32**	0.30**	0.27**	0.47**
OS6	Shortness breath	0.06	0.04	0.01	0.38**
OS7	Nausea/vomiting	0.14	0.01	0.11	**0.13**
OS8	Abdominal pain/ diarrhea	0.07	0.01	0.08	0.26**
OS9	Constipation	0.25**	0.23**	0.06	0.37**
OS10	Rashes/itchy skin	0.09	0.16	0.08	0.52**
OS11	Facial flushing	0.07	0.06	0.06	0.46**
OS12	Body Shape/appearance	0.05	0.19*	0.18*	0.33**
OS13	Lifestyle changes	0.31**	0.28**	0.34**	0.40**
OS14	Limit to activities	0.27**	0.39**	0.22**	0.51**

*Note* Correlations between each item and its designated scale are in bold type

** There was a significant at the level of 0.01. * There was a significant at the level of 0.05

The specific item data in the QLICD-OS passed the Bartlett spheroid test and the results showed that the variables were significantly correlated with KMO statistic being 0.643, indicating that factor analysis can be performed. According to eigenvalues > 1, 5 principal components were extracted for the specific module with the cumulative explained variation being 62.896%. After the Varimax rotation method, it can be seen that the first principal component included items OS3, OS4, OS5, OS14, and the variance contribution rate was 16.62%; the second principal component included OS6, OS10, OS11 with the variance contribution rate being 15.47%; the third principal component included items OS7, OS8, OS9 with the variance contribution rate being 12.11%; the fourth principal component included items OS2 and OS12 with the variance contribution rate being 9.35%; the fifth principal component included items OS1 and OS13 with the variance contribution rate being 9.34%. These 5 main components basically reflect the clinical symptoms of bone and digestive system, drug side effects, and special psychological problems of the disease in patients with osteoporosis. The structure of the scale is roughly consistent with the theoretical conception, indicating good construct validity (Table 2).

**Table 2:** Factor loadings of factor analysis on the specific module after maximum rotation of variance

Item	Principal Component (variance contribution rate%)				
	1(16.621)	2(15.472)	3(12.114)	4(9.355)	5(9.335)
OS1	0.36	-0.03	-0.07	-0.09	0.58
OS2	0.33	0.30	-0.19	-0.65	0.11
OS3	0.71	0.26	-0.06	-0.22	-0.23
OS4	0.81	0.05	-0.08	0.01	0.18
OS5	0.79	-0.12	0.02	0.11	0.20
OS6	0.07	0.65	0.14	-0.22	-0.18
OS7	-0.20	0.10	0.83	-0.10	-0.11
OS8	0.05	0.07	0.81	0.23	-0.02
OS9	0.05	0.23	0.51	-0.10	0.44
OS10	0.05	0.88	0.16	0.13	0.03
OS11	-0.03	0.83	0.02	0.19	0.22
OS12	0.14	0.26	-0.04	0.61	0.02
OS13	-0.02	-0.02	0.01	-0.08	-0.72
OS14	0.558	0.04	0.02	0.52	0.17

#### Criterion-related validity

Table 3 lists the correlation coefficients between the domain scores of QLICD-OS and SF-36, indicating that the correlation between the same and similar domains was generally higher than the correlation between different and dissimilar domains. For example, except for the low correlation coefficients of physical function, physical role, physical pain, and emotional role with general modules, the correlation coefficients between the general module of QLICD-OS and the 8 domains of SF-36 were between 0.62 and 0.65. The correlation coefficients between the specific module of QLICD-OS and the 8 domains of SF-36 were relatively low in physical roles, physical pain, emotional role, and mental health, confirming that the criterion-related validity was reasonable.

**Table 3:** Correlation coefficients between domains of QLICD-OS and SF-36 (*n* = 127)

SF-36	QLICD-OS					
	PHD	PSD	SOD	SPD	CGD	TOT
Physical function	0.24**	0.08	0.11	0.32**	0.16	0.23**
Role of physical function	0.21*	0.11	0.14	0.12	0.18*	0.19*
Body pain	0.44**	0.22**	0.24**	0.15	0.34**	0.34**
General health status	0.43**	0.57**	0.61**	0.34**	0.62**	0.64**
Vitality	0.56**	0.55**	0.53**	0.30**	0.63**	0.63**
Social function	0.60**	0.49**	0.58**	0.28**	0.63**	0.63**
Role of emotion	0.32**	0.27**	0.20*	0.15	0.30**	0.31**
Mental health	0.53**	0.62**	0.54**	0.20*	0.65**	0.63**

*Note* PHD: physical domain, PSD: psychological domain, SOD: social domain, SPD: specific domain, CGD: Core/General domain (general module), TOT: total scale

** There was a significant at the level of 0.01. * There was a significant at the level of 0.05

Specifically, the correlation coefficient between the physical function of QLICD-OS and the general health of SF-36 was 0.43; the correlation coefficient between QLICD-OS’s mental function and SF-36’s mental health was 0.62; the correlation coefficient between the social function of QLICD-OS and that of SF-36 was 0.58. The correlation coefficient between the specific module of QLICD-OS and the 8 domains of SF-36 was between 0.12 and 0.34. The correlation coefficient between the general module of QLICD-OS and the 8 domains of SF-36 was between 0.16 and 0.65. The correlation coefficient between the overall QLICD-OS and the 8 domains of SF-36 was between 0.19 and 0.64.

### Reliability

Analysis took place of the internal consistency and split-half reliability of the general module and specific module of the QLICD-OS. Except for the specific module, the internal consistency reliability of each domain was above 0.7 and the overall internal consistency reliability was 0.88. The split-half reliability was between 0.37 and 0.86 and the split-half reliability of the entire scale was 0.72. The test-retest reliability for all domains were higher than 0.80. See Table 4 in detail.

**Table 4:** Internal consistency and split-half reliability of the QLICD-OS for osteoporosis patients

Domains	Number of items	Alpha coefficient	Split-half reliability	Test-retest reliability
Physical function	9	0.70	0.45	0.89
Psychological function	11	0.85	0.86	0.96
Social function	8	0.82	0.81	0.95
Specific module	14	0.55	0.37	0.90
General module	28	0.91	0.85	0.95
Total scale	42	0.88	0.72	0.96

### Responsiveness

The results in Table 5 showed that the changes of physical function, psychological function, social function, general module, specific module and total scale before and after treatment were statistically significant (*P* < 0.05), and the SRM was 0.35–0.79. It is can be seen that the specific module domain was less responsive for SRM was lower than 0.20.

**Table 5:** Responsiveness results of the QLICD-OS for osteoporosis patients

Domains/Facets	Before treatment	After treatment	Paired t test		SRM
	*x* ± *s*	*x* ± *s*	t	*P*	
Physical function	54.18 ± 16.51	41.21 ± 13.46	9.34	< 0.001	0.79
Basic physical functions	57.43 ± 14.21	55.76 ± 10.81	1.46	0.148	0.12
Independence	54.66 ± 39.11	22.64 ± 31.27	9.53	< 0.001	0.82
Energy and discomfort	46.95 ± 14.83	39.96 ± 10.45	6.08	< 0.001	0.47
Mental function	64.24 ± 16.06	60.27 ± 14.88	4.98	< 0.001	0.35
Cognition	65.94 ± 16.94	61.71 ± 13.80	3.54	0.001	0.25
Emotion	63.22 ± 18.96	59.36 ± 17.31	4.09	< 0.001	0.20
Will and personality	66.14 ± 18.71	62.01 ± 16.99	3.70	< 0.001	0.22
Social function	70.87 ± 17.65	64.35 ± 16.24	8.07	< 0.001	0.37
Interpersonal communication	67.91 ± 21.67	58.60 ± 19.75	7.34	< 0.001	0.43
Social support	75.53 ± 17.28	72.57 ± 16.10	3.15	0.002	0.17
Social role	68.31 ± 22.37	60.63 ± 21.83	6.87	< 0.001	0.34
General module	62.90 ± 14.53	55.31 ± 12.76	9.28	< 0.001	0.52
Specific module	70.87 ± 9.92	72.02 ± 6.95	-2.68	0.009	0.17
Clinical symptoms	62.60 ± 16.58	64.11 ± 11.74	-1.41	0.161	0.09
Drug side effects	89.65 ± 12.46	91.97 ± 8.10	-2.88	0.005	0.19
special Effects on Mentality/Life	53.48 ± 17.58	54.59 ± 14.07	-1.02	0.309	0.06
Total scale	65.37 ± 11.16	60.88 ± 9.38	6.71	< 0.001	0.40

## Discussions

Based on modular approach, a Quality of Life Scale for Osteoporosis Patients (QLICD-OS) was developed by combination of the general module (QLICD-GM) in well-developed system of quality of life instruments for chronic diseases and a newly developed osteoporosis-specific module. The general module QLICD-GM including 3 domains of physical function (9 items), mental function (11 items) and social function (8 items) can be used for all various chronic diseases, and the specific module is only for osteoporosis. Up to now, the updated QLICD system includes 34 common chronic disease such as hypertension, coronary heart disease, COPD, etc [19]. . .

As far as we know, although a number of instruments have been developed for QOL in patients with osteoporosis [8–15], none of them was developed by the modular approach. In contrast, the QLICD-OS has two significant advantages over existing instruments: (1)it can compare QOL for various diseases through the generic module and capture symptoms and side effects through the specific module, showing both general and specific attributes; (2) it is of a clear hierarchy (items→ facets→ domains→ overall) so that mean scores can be computed at different levels. It can be analyzed not only at the domain (four domains) and the overall levels but also at the different facet levels (12 facets) to detect changes in detail; (3) It can be used for all type of osteoporosis (with or without fragility fractures) at any stages because the specific module includes 3 facets and different and diverse 14 items.

The general module is of core and highlighted significance for the instrument system by modular approach. There are currently two general modules for quality of life reported. One is the general module QLQ-C30 [35] of the European QLQ series. It consists of 5 functional subscales (physical, role, cognitive, emotional and social function), 3 symptom subscales (fatigue, pain, nausea, and vomiting), 1 general health status subscale and 6 single items (dyspnea, insomnia, loss of appetite, constipation, diarrhea, and financial difficulties). The other one is the general module of the FACT (Functional Assessment of Cancer Therapy) series (FACT-G), which consisted of 27 items in 5 domains including physical status (7 items), social/family status (7 items), emotional status (6 items), and functional status (7 items). These two modules were only used to determine the QOL of cancer patients, not for various chronic diseases patients. Although FACT was renamed FACIT (Functional Assessment of Chronic Illness Therapy) later [36], the general module applied FACT-G was also for cancer patients. In terms of chronic diseases, only our QLICD-GM was directly developed for patients with chronic diseases. The QOL measurement scale for specific chronic diseases could be developed on the basis of the general module, and disease-specific items could be added to fully reflect QOL of patients with specific diseases. This facilitated the comparison of the QOL among patients with complex and diverse chronic diseases.

Usually, a practical QOL should be validated on psychometric properties at least three aspects: validity, reliability and responsiveness [33, 34]. In this study, the qualitative analysis confirmed content validity. Correlation analysis showed that the item had a strong correlation with its own domains, and a weak correlation with other domains. Factor analysis showed that the components extracted from the data were consistent basically with the theoretical structure of the scale. These results confirmed good construct validity. Correlation coefficient between the similar domains of QLICD-OS and SF-36 showed reasonable criterion-related validity, with all coefficients r being greater than 0.40 exception of physical function of SF-36 and physical domain of QLICD-OS (0.24).

Our results indicated that the instrument has good reliability given Cronbach’s α coefficients above 0.70 (exception of the specific module 0.55) and test-retest correlation coefficients above 0.80. The Possible reasons for only a weak Cronbach’s alpha value of the “specific module” (0.55) are: (1) the small sample size, (2) it includes three facets of clinical symptoms, drug side effects, special effects on mentality and life, the number of items are of relative large and heterogeneity.

Responsiveness analysis (Table 5) showed that the possibility of improvement and deterioration (if any) of quality of life over time could be detected at the domain level. Comparison of the results showed that the changes of physical function, psychological function, social function, general module, specific module and total scale before and after 1 week of treatment were statistically significant (*P* < 0.05), and the SRM was 0.35-0.79. The specific module domain was less responsive; perhaps because osteoporosis was a chronic metabolic bone disease that required long-term treatment, and the patient’s hospital stay was short, the specific module was not expected to change significantly before and after treatment in a short period of time. In other words, the instrument revealed the changes of domain scores which are expected to change. Therefore, it can be inferred that the QLICD-OS could be rated as moderate responsiveness.

### Limitations of the research

QLICD-OS is also subject to various restrictions. First, Osteoporosis patients participating in the research are limited to individuals who can read and understand the questionnaire. Second, QLICD-OS is developed based on participants with Chinese cultural background. When translating QLICD-OS into languages ​​other than Chinese, the level of cultural proficiency should be carefully evaluated. In addition, the sample size of the study is not very large, which may also affect the results related to factor analysis and responsiveness.

## Conclusion

The QLICD-OS was developed by combining the general module of chronic diseases and the specific module of osteoporosis. We recommend it to be used in measuring the quality of life of Chinese patients with osteoporosis considering the Chinese cultural background and good psychometric properties (validity, reliability and responsiveness). It needs further large-scale studies to confirm psychometric properties in different settings (community etc.).

## Data Availability

All datasets collected and analyzed in this survey will be available from the corresponding author upon reasonable request.

## Abbreviations

QOL: Quality of life
ICC: intra-class correlation
SRM: Standardized Response Mean
OQLQ: Osteoporosis Quality of Life Questionnaire
JOQLQ: Japanese Osteoporosis Quality of Life Questionnaire
OPAQ: Osteoporosis Assessment Questionnaire
OFDQ: Osteoporosis Functional Disability Questionnaire
QUALEFFO: Quality of Life Questionnaire of the European Foundation for Osteoporosis
SF-36: the MOS short form health survey
QLICD-OS: Quality of Life Instruments for Chronic Diseases-Osteoporosis
QLICD-GM: Quality of Life Instruments for Chronic Diseases-General module
